# Oscillatory Nernst effect in Pt|ferrite|cuprate-superconductor trilayer films

**DOI:** 10.1038/s41598-017-05747-6

**Published:** 2017-07-13

**Authors:** Y. Shiomi, J. Lustikova, E. Saitoh

**Affiliations:** 1 Institute for Materials Research, Tohoku University, Sendai, 980-8577 Japan; 2 Spin Quantum Rectification Project, ERATO, Japan Science and Technology Agency, Aoba-ku, Sendai, 980-8577 Japan; 3 WPI Advanced Institute for Materials Research, Tohoku University, Sendai, 980-8577 Japan; 4 Advanced Science Research Center, Japan Atomic Energy Agency, Tokai, 319-1195 Japan

## Abstract

Although magnetism and superconductivity hardly coexist in a single material, recent advances in nanotechnology and spintronics have brought to light their interplay in magnetotransport in thin-film heterostructures. Here, we found a periodic oscillation of Nernst voltage with respect to magnetic fields in Pt|LiFe_5_O_8_ (Pt|LFO) bilayers grown on a cuprate superconductor YBa_2_Cu_3_O_7−*x*_ (YBCO). At high temperatures above the superconducting transition temperature (*T*_*C*_) of YBCO, spin Seebeck voltages originating in Pt|LFO layers are observed. As temperature decreases well below *T*_*C*_, the spin Seebeck voltage is suppressed and unconventional periodic voltage oscillation as a function of magnetic fields appears; such an oscillation emerging along the Hall direction in the superconducting state has not been observed yet. Dynamics of superconducting vortices pinned by surface precipitates seems responsible for the oscillatory Nernst effect.

## Introduction

Transport effects in ferromagnet|superconductor hybrid structures have attracted considerable interest recently. In *s*-wave and *d*-wave superconductors, the Cooper pair resides in a spin-singlet state, and thus the coupling of spin and charge transport is usually weak^1^. However, both superconductivity and spin polarization can unite under the right conditions at ferromagnet|superconductor interfaces. The singlet-triplet conversion of paring amplitudes at the interfaces offers tantalizing possibilities for superconducting spintronics devices in which Joule heating and dissipation are minimized^2^. Also, it was recently reported that thermally-excited quasiparticles, superpositions of electron-like and hole-like excitation with spin-1/2, exhibit a giant spin Hall effect by electric spin injection into an *s*-wave superconductor NbN^3^.

The coupling of spin and heat currents in magnetic heterostructures is also an active field of current research, and has spawned the field of spin caloritronics^4^. In normal-metal|ferromagnet hybrid structures, one spectacular example of the spin caloritronic effects is the spin Seebeck effect (SSE)^5–7^. The SSE combined with the inverse spin Hall effect (ISHE)^8^ enables the generation of a transverse electric field due to a longitudinal thermal gradient. Also in ferromagnet|superconductor hybrid structures, thermoelectric effects are expected to lead to improved devices similar to normal-state spin caloritronics. For example, very large spin-dependent thermoelectric effects were theoretically proposed by lifting the spin degeneracy of the density of states in superconductors due to magnetic proximity effects^9^.

To date, thermoelectric effects in ferromagnet|superconductor hybrids have mainly focused on the transport of Cooper pairs and quasiparticles^10–13^. However, in addition to Cooper pairs and quasiparticles, another unique carrier in superconductors is a superconducting vortex. In type-II superconductors such as high-*T*_*C*_ superconductors, magnetic flux enters the superconductors in the form of vortices above the lower critical field. The vortex comprises a small normal core and circulating supercurrents around the core. The supercurrents decay on the length about the penetration depth *λ*, which is much longer than the coherence length *ξ* for high-*T*_*C*_ cuprate superconductors (*ξ* = 0.2–3 nm and *λ* = 100–1000 nm for YBa_2_Cu_3_O_7−*x*_ (YBCO)^14^). The magnetic flux enclosed in a vortex is quantized in the unit of ${{\rm{\Phi }}}_{0}=2.07\times {10}^{-15}\,{{\rm{Tm}}}^{2}$, the flux quantum. When a heat current is applied to a vortex system, flux lines start to move along the direction of the temperature gradient and generate electromotive force in the Hall direction, known as the vortex Nernst effect^15–17^. The dynamics of superconducting vortices is thereby compatible with the geometry of SSE, and could be harmonized with spin caloritronic research^4^ to offer a new direction in transport study of ferromagnet|superconductor hybrids.

**Figure 1:**
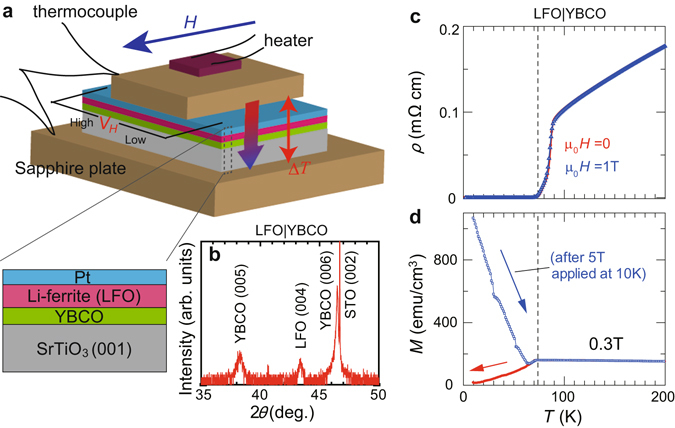
Experimental setup and samples. (**a**) Measurement setup of the longitudinal spin Seebeck effect (SSE) used in the present study. Voltage signal (*V*_*H*_) induced by a perpendicular temperature difference (Δ*T*) is measured in in-plane magnetic field (*H*). Here, Δ*T* is applied using a chip resistance heater, and is measured with a couple of type-E thermocouples. The samples are Pt|LFO|YBCO trilayers grown on STO substrates. The trilayer samples are sandwiched by two sapphire plates. (**b**) *θ*–2*θ* x-ray diffraction pattern of LFO|YBCO bilayer film grown on STO substrate. (**c**) Temperature (*T*) dependence of resistivity (*ρ*) for LFO|YBCO bilayer film in zero magnetic field (red color) and in-plane magnetic field of 1 T (blue color). The LFO|YBCO bilayer shows zero resistivity at *T*_*C*_ = 72 K. (**d**) *T* dependence of magnetization (*M*) for LFO|YBCO bilayer film measured in a field-cooling process under 0.3 T (red color) and in a field-warming process under 0.3 T after 5 T is applied at 10 K (blue color). After the strong magnetic field is applied at 10 K, the *M* value is clearly enhanced by vortex pinning.

We have investigated spin Seebeck and Nernst effects in the presence of non-equilibrium superconducting vortices in a high-*T*_*C*_ cuprate. We performed measurements of transverse thermoelectric effects for a Pt|insulating-ferrite (LiFe_5_O_8_ (LFO)) system fabricated on a film of the high-*T*_*C*_ superconductor YBCO (Fig. 1a). We chose LFO as a magnetic layer because the ferrimagnetic transition temperature of LFO is very high (around 900 K^18^), because LFO is an electric insulator below 300 K, and because the growth of LFO single-crystalline films on SrTiO_3_ (STO) substrates is well established^19^. Although YBCO is not a good spin-charge converter owing to its weak spin-orbit coupling, at low temperatures well below *T*_*C*_ of YBCO, we found periodic oscillations whose period is 0.2–0.3 T in the voltage spectra of the measurements. Since isothermal magnetization curves do not show similar oscillations, spin current scenarios are unlikely to be responsible for the voltage oscillations. We propose an oscillation mechanism of the vortex Nernst effect based on a matching effect of deformed vortex lines which are strongly pinned at the LFO interface.

## Results

### Sample characterization

Trilayer film samples of Pt|LFO|YBCO (Fig. 1a) were grown by means of pulsed laser deposition and sputtering techniques (see Methods). The LFO|YBCO bilayers deposited on STO substrates by pulsed laser deposition were characterized by x-ray diffraction as shown in Fig. 1b. The LFO|YBCO films were found to crystallize with the (001) oriented perovskite structure. On these LFO|YBCO films, 10-nm-thick Pt films were deposited by sputtering for spin Seebeck and Nernst effects measurements described later. Two Pt|LFO|YBCO samples (Sample #1 and Sample #2) were used in the transverse thermoelectric measurements (see Methods). For control experiments, the same measurements were performed for YBCO, Pt|YBCO, Pt|LFO, LFO|YBCO, and Pt|STO|YBCO films grown by the same method.

### Superconductivity in YBCO layer

Resistivity, *ρ*, for the LFO|YBCO bilayer film measured without applying a magnetic field is shown as a function of temperature (*T*) in Fig. 1c. *ρ* decreases with decreasing *T* from 200 K almost in proportion to *T* and shows a clear drop around 90 K. The occurrence of the zero resistivity state below 72 K clearly demonstrates that the YBCO layer shows superconductivity below that temperature (*T*_*C*_ = 72 K). The zero resistivity state is also observed in strong magnetic fields (see also Supplementary Information). As shown in Fig. 1c, the *ρ*-*T* curve measured under *μ*_0_ *H* = 1 T is almost the same as that in zero magnetic field.

The superconducting transition of the YBCO layer is also observed in magnetization measurements. Figure 1d shows *T* dependence of the magnetization *M* for LFO|YBCO at *μ*_0_ *H* = 0.3 T. In the *T* region where the YBCO layer is in the normal state, ferrimagnetic magnetization of the LFO layer, which is about 160 emu/cm^3^ $\approx$ 3.3 *μ*_*B*_/LiFe_5_O_8_ and almost independent of *T*, is observed. In a field-cooling process under 0.3 T, the magnetization starts to decrease at *T*_*C*_ because of the diamagnetism of the superconducting YBCO layer. However, after a strong magnetic field of 5 T is applied at 10 K, the magnetization value changes significantly, as shown in Fig. 1d. The magnetization in the YBCO layer increases due to the pinning effect on superconducting vortices. The magnitude of the net magnetization at the lowest *T* under 0.3 T is more than five times greater than that in the normal state. Since the pinning force for vortices is weaker at higher temperatures, *M* decreases monotonically as *T* is raised towards *T*_*C*_.

### Spin Seebeck and Nernst effects for Pt|LFO|YBCO trilayer film

**Figure 2:**
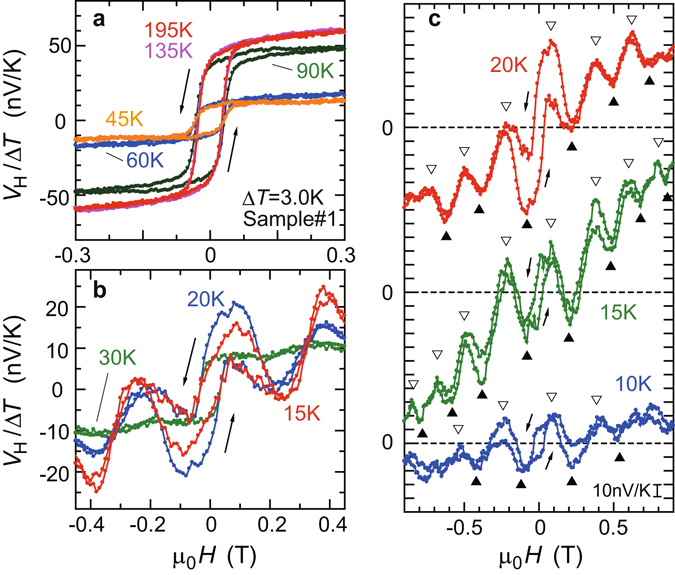
Periodic voltage oscillations in transverse thermoelectric effects for the Pt|LFO|YBCO #1 sample. Magnetic field (*H*) dependence of *V*_*H*_/Δ*T* for the Pt|LFO|YBCO #1 sample (Sample #1) (**a**) at high temperatures and (**b**,**c**) at low temperatures. The Δ*T* value was set to be 3.0 K. In (**c**) *V*_*H*_/Δ*T* at 10 K, 15 K, and 20 K is shown in a broad magnetic-field range up to 0.9 T. As shown in (**b**,**c**) in the low temperature regime below 30 K, periodic voltage oscillations are clearly observed in *V*_*H*_/Δ*T*. As highlighted by triangles in (**c**) the periodic oscillations persist up to 0.9 T.

**Figure 3:**
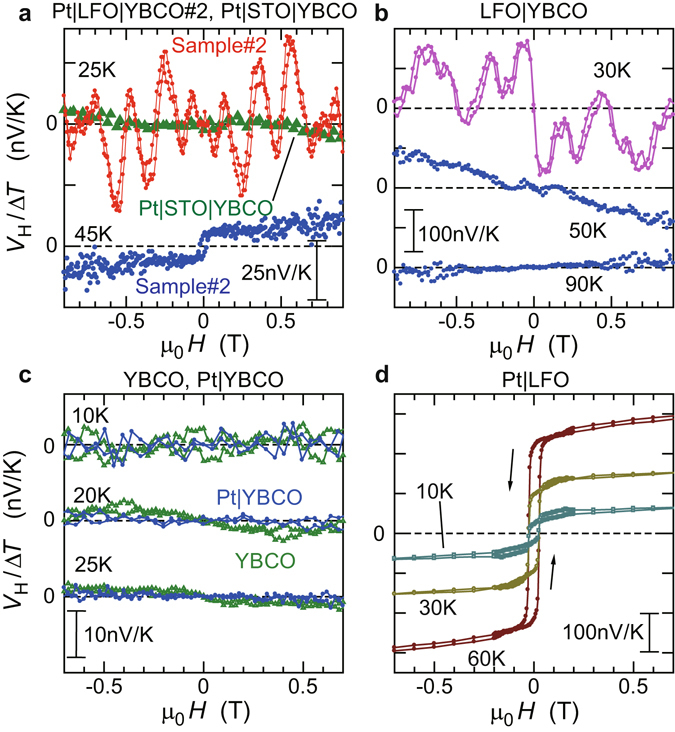
Reproducibility and control experiments. Magnetic field (*H*) dependence of *V*_*H*_/Δ*T* for (**a**) the Pt|LFO|YBCO #2 sample (denoted by Sample #2) and Pt|STO|YBCO, (**b**) LFO|YBCO, (**c**) YBCO and Pt|YBCO, and (**d**) Pt|LFO at several temperatures. For the Pt|LFO|YBCO #2 sample (Sample #2) in (**a**), clear periodic oscillations are observed at 25 K, while only SSE voltage in the Pt|LFO layer is observed at 45 K. In Pt|STO|YBCO in (**a**), YBCO in (**c**), Pt|YBCO in (**c**), or Pt|LFO in (**d**), oscillation effects are not observed at any temperature. Oscillations of *V*_*H*_ are also observed for LFO|YBCO at 30 K in (**b**).

We employ the longitudinal SSE measurement setup^20^ for Pt|LFO|YBCO samples with spin current along the out-of-plane temperature gradient, as illustrated in Fig. 1a. Figure 2 shows the *H* dependence of *V*_*H*_/Δ*T* for the Pt|LFO|YBCO #1 sample (Sample #1) at various temperatures. In Fig. 2a, above *T*_*C*_, voltage signals which trace the magnetization curves of the insulating LFO layer are observed. These signals are attributed to the spin Seebeck voltage in the Pt|LFO layer, since similar signals of the same sign were observed in bilayer Pt|LFO samples (Fig. 3d) and since ISHE in YBCO is negligibly small owing to its weak spin-orbit coupling (Fig. 3b). Below *T*_*C*_, the magnitude of the spin Seebeck voltage largely decreases, as observed at 45 K and 60 K in Fig. 2a. Because the total resistance of the Pt|LFO|YBCO trilayer is steeply lowered as the YBCO layer shows superconductivity, electric shunting reduces the magnitude of the spin Seebeck voltage originating in the Pt|LFO layer below *T*_*C*_.

As *T* further decreases, a novel voltage oscillation is observed in the voltage spectra. Figure 2b shows the *H* dependence of *V*_*H*_/Δ*T* at 30 K, 20 K, and 15 K. At 30 K, small oscillations are observed in the spin Seebeck voltage above 0.1 T. This oscillation becomes more visible at lower temperatures, and periodic oscillations of *V*_*H*_ are clearly observed at 20 K and 15 K, as shown in Fig. 2b. *V*_*H*_ even shows a sign change around 0.2 T at 15 K, which has not been observed in any SSE experiments for normal-metal|magnet bilayers. The *V*_*H*_ data in the low *T* range is shown up to 0.9 T in Fig. 2c. The periodic oscillations persist in the high-*H* range. The period of oscillations is 0.3 T and is almost independent of *T*.

### Reproducibility and control experiments

We confirmed that similar voltage oscillations are observed also in another sample, the Pt|LFO|YBCO #2 sample (Sample #2) at 25 K in Fig. 3a. The period of the oscillations is about 0.2 T, which is similar to that observed in Sample #1. Moreover, the voltage oscillations are no longer observed at 45 K for Sample #2, also consistent with the results at 45 K in Sample #1. At 45 K, only SSE voltage originating in the Pt|LFO layer is observed for Sample #2, as shown in Fig. 3a.

For control experiments, we have also conducted similar measurements of spin Seebeck and Nernst effects in YBCO, Pt|YBCO, Pt|LFO, LFO|YBCO, and Pt|STO|YBCO samples. As shown in Fig. 3a–d, the oscillation effects are never observed in YBCO, Pt|YBCO, Pt|LFO, or Pt|STO|YBCO samples at any temperatures. In contrast, oscillatory *H* dependence is also observed in LFO|YBCO at 30 K in Fig. 3b, whereas the periodicity of this oscillation in LFO|YBCO is rather complex compared to the Pt|LFO|YBCO samples (see Figs 2b,c and 3a). These results indicate that the LFO|YBCO bilayer structure is necessary for oscillations to appear in the spin Seebeck measurement.

### Pinning of superconducting vortices

**Figure 4:**
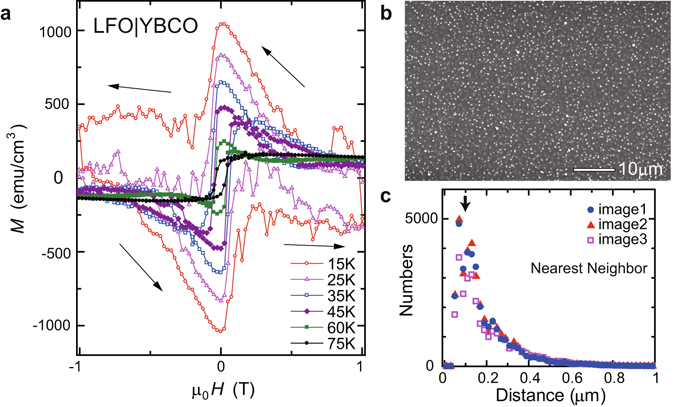
Vortex magnetization and surface pinning. (**a**) Magnetic field (*H*) dependence of magnetization (*M*) for LFO|YBCO bilayer film at selected temperatures. Below *T*_*C*_, magnetization is enhanced by vortex pinning in the YBCO layer. (**b**) SEM image of the surface of YBCO film. Numerous white points indicate surface precipitates, which act as pinning sites of vortices. (**c**) Distance distribution of nearest-neighbor surface precipitates observed in the SEM images. To ensure the reliability of analysis, the distance distribution was estimated for three SEM images (image1, image2, and image3) taken at different places of the YBCO surface. For all the images, a peak is observed at ~0.1 *μm* as highlighted by the arrow, which indicates that the surface pinning sites of superconducting vortices have a spatial correlation length of 0.1 *μm* on average.

To examine the magnetic properties of the LFO|YBCO bilayer at low temperatures, an *M*-*H* measurement under in-plane magnetic fields was performed for LFO|YBCO, as shown in Fig. 4a. At 75 K (>*T*_*C*_), the *M*-*H* curve reflects the magnetization process of the ferrimagnetic LFO layer. With decreasing *T* below *T*_*C*_, however, the magnetization curves become dramatically different from those above *T*_*C*_ owing to the vortex magnetization in the YBCO layer. Below *T*_*C*_, vortex pinning in the layered CuO_2_ planes (intrinsic pinning) and also in other extrinsic pinning sites enhances the magnetization. At 25 K and 15 K, the magnetization process of the LFO layer is hard to recognize and giant hystereses are observed up to 1 T; these large hystereses in the magnetization curves are in stark contrast with almost no hystereses in the *V*_*H*_ signals. At very low temperatures where the periodic oscillation effect was observed in the transverse thermoelectric measurement, the ferrimagnetic magnetization in the LFO layer and the vortex magnetization in the YBCO layer can be coupled strongly. No oscillations, however, are observed in the magnetization curves, whereas flux jumps^21, 22^ are suggested from the observed noisy magnetization spectra in the very low-*T* range.

In the low temperature regime where the pinning of superconducting vortices is significant, surface precipitates formed on the YBCO layer are known to be major pinning sites^23^. As shown in a scanning electron microscope (SEM) image of the YBCO surface in Fig. 4b, numerous precipitates are found; in fact, in YBCO films grown by virtually all vapor deposition methods, nucleation of surface precipitates of non-superconducting phases has been reported^24^. These surface precipitates strongly trap superconducting vortices^23^ near the YBCO surface at very low temperatures, since thermal activation of vortices over the pinning potential is smaller at lower temperatures. As shown in Fig. 4c, the distance distribution for nearest-neighbor precipitates has a clear maximum at 0.1 *μm* (see the arrow in Fig. 4c). Hence, surface pinning sites of superconducting vortices can be spatially correlated with the characteristic distance of 0.1 *μm* on average.

### Underlying physical mechanism

To date, various oscillation effects as a function of external magnetic fields were reported in YBCO, *e*.*g*. oscillatory critical current density^25, 26^, oscillatory magnetization^27^, oscillatory mangetoresistance^28–30^, and oscillatory vibration damping^31, 32^. However, a critical difference of the present effect from the reported oscillation effects^25–32^ is that the voltage oscillation (Figs 2 and 3) emerges along the Hall (Nernst) direction, while all of the known oscillations effects^25–32^ are even functions with *H*. Also, in contrast to the oscillatory mangetoresistance^28–30^, the voltage oscillation is observed in the zero-resistivity state well below *T*_*C*_ (Fig. 1c). Although an oscillatory magnetoresistance with a magnetic-field period of ~0.1 T was observed in YBCO films at 71.0–74.1 K in the low-field range below 1 T^29^, our sample exhibits the zero-resistivity state up to 9 T below 30 K where the voltage oscillation was observed (see also Fig. S3 in Supplementary Information). Furthermore, the oscillation period in our case is not determined by the sample cross-sectional area transverse to the magnetic field or the distance between the CuO_2_ planes (~1 nm) in contrast to the previous studies^25–28, 30–32^. The oscillation periods in the previous papers^25–28, 30–32^ were determined by ${{\rm{\Phi }}}_{0}/S$, where *S* is the effective cross sectional area facing the magnetic field. In our Pt|LFO|YBCO samples, however, from the dimension of the YBCO layer and the distance between the CuO_2_ planes, the expected period (${{\rm{\Phi }}}_{0}/S$) is estimated to be 0.01–1 mT, which is much less than the observed oscillation period (~0.2 T). Hence, the mechanisms of these effects^25–32^ cannot be applied straightforwardly to the data presented here.

As to the physical mechanism responsible for the observed periodic oscillations, let us first consider a possible ISHE contribution induced by SSE in the superconducting YBCO layer. As reported in an *s*-wave superconductor NbN^3^, thermally-excited quasiparticles can produce ISHE voltage in the superconducting state, although further experimental verification in other *s*-wave superconductors seems necessary^1^. In our case, however, ISHE mediated by quasiparticles does not explain the oscillations, since the YBCO length along the voltage contacts is much greater than the charge imbalance length, the length scale where the spin-polarized quasiparticle currents relax into the Cooper-pair condensates^3^. Also, the quasiparticle scenario does not explain the presence of the onset temperature of the oscillations (~30 K), which is much lower than *T*_*C*_. Furthermore, the different *H* dependences of the oscillatory *V*_*H*_ signals (Figs 2b,c and 3a) and bulk magnetization (Fig. 4a) at low temperatures do not reconcile with previously established mechanisms for SSE in normal-metal|ferromagnet bilayers^33–35^.

**Figure 5:**
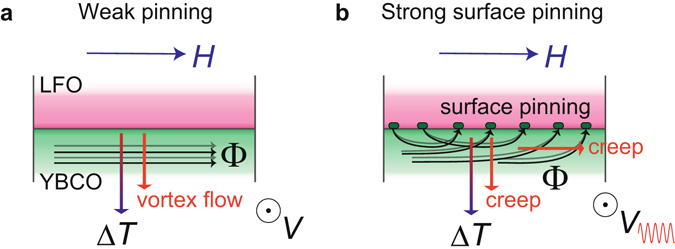
Possible mechanism of oscillatory Nernst effect. (**a**) A schematic illustration of conventional vortex Nernst effect. At relatively high temperatures below *T*_*C*_, vortex pinning is weak. Then, the vortex flow along the Δ*T* direction induces Nernst voltage along the direction perpendicular to both ${\rm{\Phi }}$ and Δ*T* directions. (**b**) At very low temperatures, surface pinning potential is so strong that vortices cannot flow freely inside the YBCO layer. In this case, creep (deformation) of trapped vortices driven by Δ*T* is expected to induce Nernst voltage. Since superconducting vortices are rearranged at different *H* values, a matching effect is expected to occur if the averaged distance between vortices matches that of surface pinning sites (0.1 *μm*).

As opposed to the suppression of thermal excitations of quasiparticles at lower temperatures, the number of vortices pinned in the YBCO layer increases monotonically with decreasing *T*, as indicated in Figs 1d and 4a; the vortices can play a more important role at lower temperatures. Since the vortex Nernst effect (Fig. 5a) was observed in various superconductors^15^, the Nernst voltage due to a thermally induced flow of vortices can be responsible for the observed periodic oscillations in *V*_*H*_ spectra (Figs 2b,c and 3a). When the temperature is low enough below *T*_*C*_, however, superconducting vortices are strongly pinned inside YBCO, and hence the Nernst signals due to a vortex flow should vanish at very low temperatures^14^. In fact, it was reported that the vortex Nernst signal for YBCO below 1 T shows a maximum just below *T*_*C*_ but disappears below 50 K due to the strong pinning effects^36^. In the transverse thermoelectric experiment for LFO|YBCO in Fig. 3b, a relatively large *H*-linear Nernst signal probably due to the vortex Nernst effect is observed at 50 K; the sign change in the slope of *V*_*H*_ between 50 K (below *T*_*C*_) and 90 K (above *T*_*C*_) in Fig. 3b is consistent with the reported *T*-dependence of the Nernst coefficient^36^. Hence, the periodic voltage oscillations in Pt|LFO|YBCO are observed in the low temperature regime where pinning of superconducting vortices is so strong that the vortices cannot flow along the Δ*T* direction.

One plausible mechanism of the observed oscillations in Figs 2b,c and 3a,b is a Nernst effect induced by vortex creep, *i*.*e*. Δ*T*-driven deformation of vortex lines near the LFO|YBCO interface, as illustrated in Fig. 5b. It was reported that the creep rate of vortices in YBCO reaches a maximum at 20–25 K^37^, which is close to the onset temperature of the observed oscillations. As discussed in the previous section, in the very low-temperature range, superconducting vortices are pinned by surface precipitates. Since pinning of vortices in the superconducting layer is enhanced in magnet|superconductor bilayers^38^, the pinning of vortices near the YBCO surface can be stronger in LFO|YBCO than in the YBCO single-layer film thanks to the LFO magnetization. In spite of the surface pinning, application of a temperature difference across the sample thickness can induce vortex creep near the LFO|YBCO interface. The deformation of vortex lines along the perpendicular-to-plane and also in-plane directions (Fig. 5b) gives rise to Nernst voltages in the YBCO layer (Fig. 5b).

When the external magnetic field is swept in the measurements of transverse thermoelectric effects for Pt|LFO|YBCO and LFO|YBCO, the vortices trapped near the LFO|YBCO interface are rearranged at different *H* values, to minimize the energy of the repulsive vortex-vortex interaction and the surface pinning potential. Since the surface pinning sites have a spatial correlation length of 0.1 *μm* (Fig. 4c), the average vortex arrangement near the interface should be periodic. The averaged pinning should be most significant at the *H* values where the averaged distance between vortices matches that of surface pinning sites (0.1 *μm*). This hypothesis is supported by the fact that the period of vortex arrangement *a* ~ 0.1 *μm* corresponds to the matching periodicity of ${B}_{p}\approx {{\rm{\Phi }}}_{0}/{a}^{2}=0.2\,{\rm{T}}$; this *B*_*p*_ value is well consistent with the oscillation periods observed experimentally in Figs 2b,c and 3a. In the matching conditions, the creep velocity which is due to thermal activation over the pinning barrier^39^ shows minimal values, and also the creep suffers strong disturbance due to the pinning force^40^. Thus, the *H* dependence of the Nernst voltage is expected to be modulated with the period of *B*_*p*_. The oscillatory Nernst effect observed in the present experiments for YBCO can thus be explained by the competition between vortex pinning and vortex creep in superconductor|ferrimagnet bilayers.

## Methods

### Sample growth

YBCO and LFO thin films were grown on 0.5-mm-thick STO (001) substrates by pulsed laser deposition from polycrystalline targets using a KrF excimer laser. The targets were made by a conventional solid state reaction method in air. During the laser ablation, oxygen partial pressure was kept at 0.3 torr and substrate temperature was at 700 °C. After deposition, the samples were annealed at 700 °C in the 400 torr oxygen atmosphere and then cooled to room temperature. The thickness of each layer is 36 nm for the YBCO layer and 60 nm for the LFO layer. A 10-nm-thick top Pt electrode film was then prepared by sputtering at room temperature in an Ar atmosphere *ex situ* on LFO|YBCO bilayers.

### Resistivity and magnetization measurement

Resistivity of LFO|YBCO was measured using a conventional 4-wire resistance method in a superconducting magnet. Ohmic contacts were made on the LFO|YBCO sample using indium solder. Magnetization measurements for LFO|YBCO were performed using the RSO technique of a SQUID magnetometer (Magnetic Property Measurement System; Quantum Design, Inc.).

### Measurement of spin Seebeck and Nernst effects

Measurements of transverse thermoelectric effects using the longitudinal SSE measurement setup^20^ were performed in a superconducting magnet (Physical Property Measurement System; Quantum Design, Inc.), where an external magnetic field (*H*) was applied parallel to the film planes. The sample was sandwiched by two sapphire blocks. The temperature difference (Δ*T*) between the two sapphire blocks was applied using a chip resistance heater and measured with a couple of type-E thermocouples (Fig. 1a)^41^. The Δ*T* value was typically set to be 2.0–4.0 K. The transverse thermal voltage (*V*) which arises in a perpendicular direction to both Δ*T* and *H* directions was measured using a nanovoltmeter at each *H* value. The *H*-odd components of induced voltage signals were extracted by ${V}_{H}(H)=\{V(H)-V(-H)\}/2$. The Pt|LFO|YBCO #1 sample (Sample #1) was 6 mm long along the direction of voltage detection and 2 mm wide along the *H* direction, and the #2 sample (Sample #2) 6 mm long and 3 mm wide. To make voltage electrodes on the samples, a silver conductive paste was used for Pt|LFO|YBCO, Pt|STO|YBCO, YBCO, Pt|YBCO, and Pt|LFO, while indium solder was used for LFO|YBCO.

## Electronic supplementary material

Supplementary Information
