# Supplementary material for: Oscillatory Nernst effect in Pt|ferrite|cuprate-superconductor trilayer films
Source: Sci Rep. 2017 Jul 13;7:5358. doi: 10.1038/s41598-017-05747-6 (PMC5509755; doi:10.1038/s41598-017-05747-6)
Supplement: Supplementary file 1 — Supplementary Information [file 41598_2017_5747_MOESM1_ESM.pdf]

Supplementary Information for "Oscillatory Nernst effect in Pt|ferrite|cuprate-superconductor trilayer films"

Y. Shiomi, J. Lustikova, and E. Saitoh

1. ΔT dependence of Nernst voltage

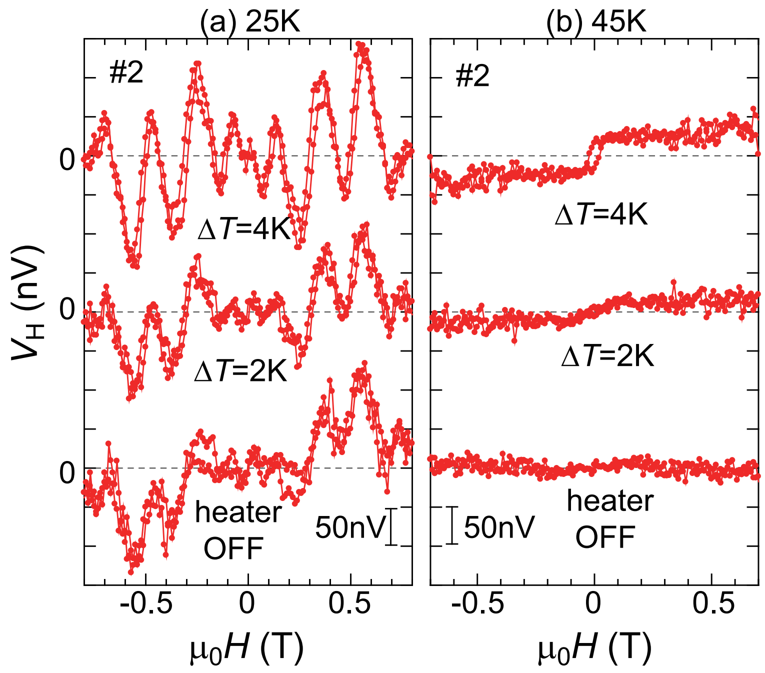

FIG. S1: ΔT **dependence of Nernst voltage.** Magnetic field (H) dependence of Nernst voltage measured at (a) 25 K and (b) 45 K for the Pt|LFO|YBCO #2 sample (#2). The ΔT value is 4 K, 2 K, or nominally 0 (heater off). Thermoelectric voltage is almost zero in the heater-off condition at 45 K, while voltage oscillation remains even in the heater-off condition ($\Delta T \approx 0$) at 25 K; note that there remains the periodic voltage oscillation of ~ 0.2 T after the data in the heater-off condition is subtracted from that measured at $\Delta T = 2$ K or 4 K. Since vortex distribution is spatially inhomogeneous along the perpendicular direction because of the dominant interface pinning, vortices can creep by tiny residual temperature difference (1-10 mK).

2. Magnetic-field angular dependence of Nernst voltage

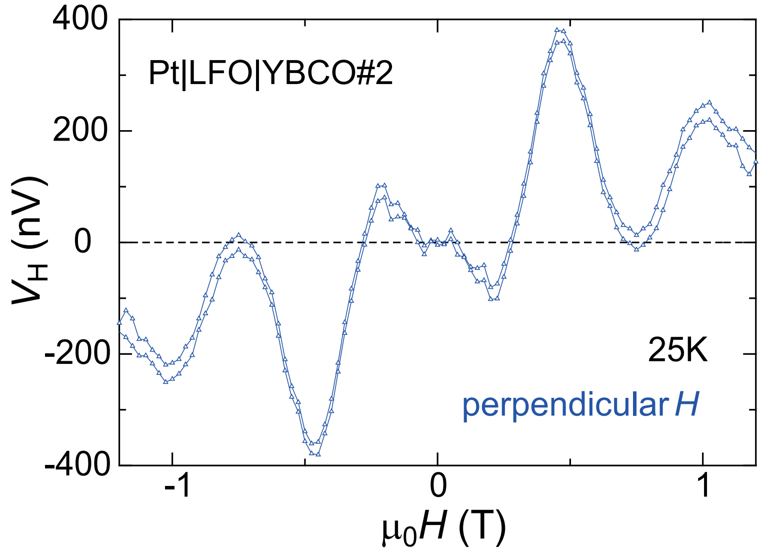

FIG. S2: **Magnetic-field angular dependence of Nernst voltage.** Magnetic field (H) dependence of Nernst voltage measured at 25 K for the Pt|LFO|YBCO #2 sample in perpendicular-to-plane H configuration. The periodic oscillation is observed also in perpendicular-to-plane magnetic fields. The presence of the voltage oscillation under perpendicular-to-plane magnetic fields indicates complicated arrangement of vortices in the YBCO layer, which seems consistent with the scenario of Nernst voltage induced by creep of interface-pinned vortices (see Fig. 5**b** in the main text).

3. Temperature dependence of resistivity under strong magnetic fields

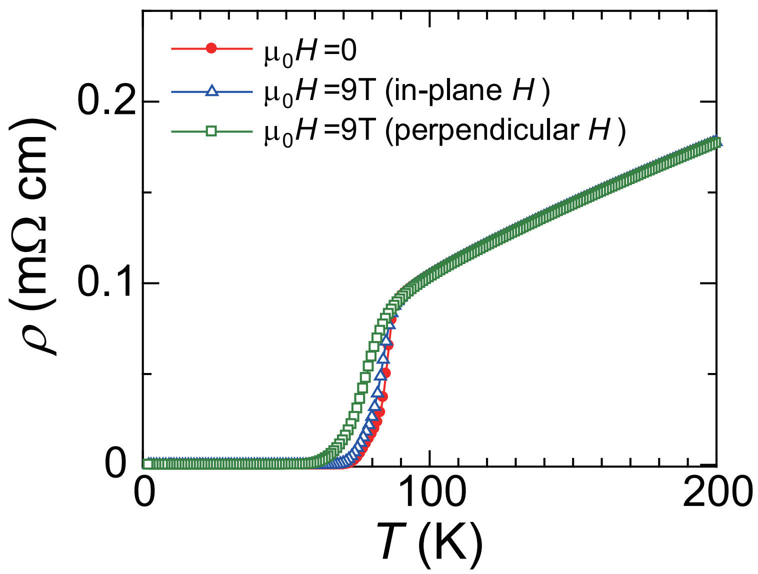

FIG. S3: **Temperature dependence of resistivity under strong magnetic fields.** Temperature (T) dependence of resistivity (ρ) for LFO|YBCO bilayer film in zero magnetic field (red color), in-plane magnetic field of 9 T (blue), and perpendicular-to-plane magnetic field of 9 T (green). Even under 9 T, the zero resistivity state was observed below $\sim$ 60 K.
